# Exosome-derived ENO1 regulates integrin α6β4 expression and promotes hepatocellular carcinoma growth and metastasis

**DOI:** 10.1038/s41419-020-03179-1

**Published:** 2020-11-12

**Authors:** Keqiu Jiang, Chengyong Dong, Zeli Yin, Rui Li, Jiakai Mao, Chengye Wang, Junlin Zhang, Zhenming Gao, Rui Liang, Qi Wang, Liming Wang

**Affiliations:** 1 Engineering Research Center for New Materials and Precision Treatment Technology of Malignant Tumors Therapy, Dalian Medical University, NO. 467 Zhongshan Road, Dalian, Liaoning 116027 China; 2 Engineering Technology Research Center for Translational Medicine, Dalian Medical University, NO. 467 Zhongshan Road, Dalian, Liaoning 116027 China; 3 Division of Hepatobiliary and Pancreatic Surgery, Department of General Surgery, The Second Affiliated Hospital of Dalian Medical University, NO. 467 Zhongshan Road, Dalian, Liaoning 116027 China; 4 Department of Respiratory Medicine, The Second Affiliated Hospital of Dalian Medical University, NO. 467 Zhongshan Road, Dalian, Liaoning 116027 China

**Keywords:** Liver cancer, Metastasis

## Abstract

Alpha-enolase (ENO1) has been found to be dysregulated in several human malignancies, including hepatocellular carcinoma (HCC). Although the role of ENO1 as a glycolytic enzyme in HCC cells has been well characterized, little is known about the other roles of ENO1, especially exosome-derived ENO1, in regulating HCC progression. Here, we demonstrated that ENO1 is frequently upregulated in HCC cells or tissues, with even higher expression in highly metastatic HCC cells or metastatic tissues as well as in exosomes derived from highly metastatic sources. Moreover, ENO1 expression is associated with the tumor-node-metastasis (TNM) stage, differentiation grade and poor prognosis in HCC patients. Surprisingly, ENO1 can be transferred between HCC cells via exosome-mediated crosstalk, exhibiting an effect similar to that of ENO1 overexpression in HCC cells, which promoted the growth and metastasis of HCC cells with low ENO1 expression by upregulating integrin α6β4 expression and activating the FAK/Src-p38MAPK pathway. In summary, our data suggest that exosome-derived ENO1 is essential to promoting HCC growth, metastasis, and further patient deterioration. The findings from this study implicate a novel biomarker for the clinical evaluation of HCC progression, especially the prediction of HCC metastatic risk.

## Introduction

Liver cancer is the fourth most common cause of cancer-related death and is the second most lethal type of cancer, with a 5-year survival rate of 18%^1^. Hepatocellular carcinoma (HCC), a main type of primary liver cancer, usually occurs in patients with hepatitis virus infection, alcohol abuse, metabolic syndrome or obesity. Because of the high incidence of metastasis, systemic treatment is highly recommended for a large proportion of HCC patients. However, the refractory nature of HCC, possible hepatic dysfunction, and the diversity of the patient population limit the use of systemic treatment^2^. Rapid deterioration and death remain the ultimate outcomes of these patients. Therefore, revealing the mechanism underlying HCC growth and metastasis, and identifying risk factors that can predict HCC metastasis will provide important scientific evidence for uncovering more therapeutic targets and improving clinical outcomes.

Exosomes (50–150 nm in diameter), a class of extracellular vesicles, are nanosized vesicles surrounded by bimolecular lipid membranes and are formed by a series of precise regulatory processes, such as the “endocytosis-fusion-efflux” process. Exosomes usually contain a variety of biomolecules, including proteins, nucleic acids, lipids, and even viruses^3,4^. The role of exosomes and their contents as potential contributors to oncogenesis and tumor metastasis is a current research focus. Notably, tumor cells can release significantly more exosomes than healthy cells, and the contents of tumor cell-derived exosomes differ from those of healthy cell-derived exosomes^5,6^. This difference not only reflects the selective regulation of tumor-derived exosomes (TEXs), based on their structure and function, but also suggests that TEXs facilitate tumor growth and metastasis via exosome-mediated crosstalk. Proteins are the executors of physiological and pathological functions and are also a critical cargo in TEXs. On the one hand, certain specific proteins in TEXs can be transferred to recipient cells to regulate important biological processes such as tumor proliferation, metastasis, and immune escape^7–11^. On the other hand, some proteins in TEXs can partially reflect the dysregulated protein profile in tumor cells, reveal the characteristics of tumor cells, and provide a new direction for the diagnosis or prognostic evaluation of tumors^12,13^. Numerous recent studies have confirmed that exosomes play a vital role in the progression of HCC^14–18^.

Alpha-enolase (ENO1), one of the three major enolases, is a key regulatory enzyme in glycolysis and is widely present in various cells and tissues^19,20^. In addition to its well-known enzymatic function during glycolysis, ENO1 has multiple functions and differential expression depending on its localization and the pathophysiological, metabolic or growth state^20–22^. Emerging evidence has demonstrated that, in addition to participating in the Warburg effect to promote glucose uptake and lactic acid production by tumor cells^23,24^, ENO1 is also directly involved in tumor cell division, proliferation, apoptosis and metastasis; immune regulation; and chemotherapeutic resistance^25–28^. Moreover, many proteomics studies based on TEXs have shown that ENO1 is selectively enriched in some TEXs, and ENO1 has thus become a potential biomarker for tumor detection^29–31^. Although the special functions of ENO1 in some tumors have been well revealed, the role of exosome-derived ENO1 in the regulation of tumor progression, particularly in HCC, is incompletely clarified.

With the emergence of exosome education philosophy, in this study, HCC cells were cultured in medium supplemented with designated HCC cell-derived exosomes (50 μg/ml) in a process that we defined as ‘education’^32^. Based on the heterogeneous metastatic potential of different HCC cells, we clarified the effect of highly metastatic HCC cell-derived exosome education. Further studies showed that ENO1 is specifically enriched in highly metastatic HCC cells and exosomes derived from these cells and is significantly correlated with poor prognosis in HCC. Subsequently, the effect of exosome-derived ENO1 on the proliferation and metastasis of HCC cells was evaluated in vitro and in vivo, revealing that exosome-derived ENO1 promotes HCC progression by regulating the expression of integrin α6β4 and the activation of downstream signaling pathways. The findings of this study indicated that ENO1 not only functions as an oncogene in HCC cells but also can be transferred to other HCC cells via exosome-mediated crosstalk, further promoting HCC growth and metastasis. The findings from this study implicate a novel biomarker for the clinical evaluation of HCC progression, especially the prediction of HCC metastatic risk.

## Materials and methods

### Cell lines and cell culture

The human HCC cell line HepG2 was obtained from the American Type Culture Collection (ATCC) and cultured in MEM (KeyGEN BioTECH, China) supplemented with 10% FBS (Gibco, USA) in a humidified incubator with 5% CO_2_ at 37 °C. The human normal liver cell line LO2 and the human HCC cell lines MHCC97L, MHCC97H, and HCCLM3 were obtained from the Cell Bank of the Chinese Academy of Sciences and cultured in DMEM (KeyGEN BioTECH) supplemented with 10% FBS in a humidified incubator with 5% CO_2_ at 37 °C. All cell lines were authenticated by the short tandem repeat (STR) profiling and tested for mycoplasma contamination. Exosome-depleted FBS was obtained by ultracentrifugation at 100,000 × *g* overnight at 4 °C. All cells ready for exosome isolation were cultured in medium supplemented with 10% exosome-depleted FBS in a humidified incubator with 5% CO_2_ at 37 °C.

### Cell proliferation assay

Cells were seeded into 96-well plates (Jet Bio-Filtration, China) in triplicate at a density of 1000 cells per well. Cell viability was assessed by Cell Counting Kit-8 (CCK-8) assay (KeyGEN BioTECH) according to the manufacturer’s instructions. The optical density (OD) at 450 nm was measured at the indicated time points using a multifunctional microplate reader (Thermo Fisher Scientific, USA). Cell growth curves were generated using GraphPad Prism software (GraphPad Software, USA).

### Colony formation assay

Cells were seeded into 6-well plates (Jet Bio-Filtration) in triplicate at a density of 2000 cells per well and cultured for 2 weeks. Subsequently, the cells were washed three times with PBS (HyClone, USA) and stained with crystal violet staining solution (KeyGEN BioTECH), followed by photoimaging under a microscope (Olympus, Japan). Colony formation rate = (number of colonies containing at least 50 cells/number of inoculated cells) × 100%.

### Wound healing assay

Cells were seeded into 6-well plates in triplicate at a suitable density. When the cells grew to 80–90% confluence, wounded areas approximately 500-μm wide were created, and floating cells were removed. The cells were then cultured in serum-free medium for another 24 h. The process of cell migration to cover the wounded area was visualized and photoimaged under a microscope every 6 h. Data were analyzed using ImageJ software (NIH, USA).

### Transwell migration and invasion assays

Cells were seeded into Transwell chambers (Corning, USA) in triplicate at a density of 5 × 10^4^ cells per well in 200 μl of serum-free culture medium. For cell invasion assays, the Transwell membranes were precoated with Matrigel (BD, USA) before cell seeding. Then, 600 μl of culture medium supplemented with 20% FBS was added into the lower compartment of the Transwell chambers as a chemoattractant. The cells were allowed to migrate for 24 h or invade for 48 h and were then fixed with 4% paraformaldehyde (KeyGEN BioTECH) for 30 min and stained with crystal violet staining solution for 10 min. Crystal violet-stained cells adhering to the lower surface of the Transwell membranes were counted under a microscope in five random fields. Data were analyzed using ImageJ software.

### Immunofluorescence (IF) staining

Cells were seeded in 6-well plates containing three cover slides at a density of 5000 cells per well and cultured overnight. The cells were then fixed with 4% paraformaldehyde for 30 min, permeabilized with 0.2% Triton X-100 (Sigma, USA) for 5 min and blocked with 2% BSA (Sigma) for 1 h at room temperature. Subsequently, the cells were incubated with primary antibodies against E-cadherin (1:100, Proteintech, 20874-1-AP, USA), N-cadherin (1:100, Proteintech, 22018-1-AP), Vimentin (1:100, Proteintech, 10366-1-AP), integrin α6 (1:1000, Abcam, ab20142, UK) and integrin β4 (1:500, Abcam, ab133682) separately overnight at 4 °C. The next day, the cells were incubated with Alexa Fluor 488-conjugated secondary antibody (1:100, Proteintech, SA00013-1/ SA00013-2) or Alexa Fluor 594-conjugated secondary antibody (1:100, Proteintech, SA00013-3/SA00013-4) for 1 h at room temperature in the dark. Cell nuclei were counterstained with DAPI (Sigma) for 5 min at room temperature in the dark. IF signals were visualized using an Olympus IX81 fluorescence microscope (Olympus) and a Leica TCS SP5II confocal microscope (Leica, Germany). Data were analyzed using ImageJ software.

### SDS-PAGE and western blot analysis

Total cellular and exosomal proteins were obtained using a RIPA lysis buffer (KeyGEN BioTECH) containing a protease inhibitor cocktail (Sigma) and a phosphatase inhibitor cocktail (Sigma) for use in further assays. A bicinchoninic acid (BCA) assay kit (Thermo Fisher Scientific) was used to measure protein concentrations. Equal amounts of cell lysate or exosome lysate were resuspended in 5× loading buffer (KeyGEN BioTECH) and were subsequently incubated at 95 °C for 5 min and then centrifuged in a microcentrifuge at 15,000 × *g* for 5 min. Samples were separated on 10% SDS-PAGE gels (KeyGEN BioTECH) and transferred to nitrocellulose membranes (Millipore, USA). Membranes were stained with Ponceau red staining solution (KeyGEN BioTECH) and were then washed with TBS containing 0.1% Tween 20 (TBS-T). The destained membranes were blocked with 5% nonfat milk (BD) for 1 h at room temperature. Then, primary antibodies against E-cadherin (1:5000, Proteintech, 20874-1-AP), N-cadherin (1:2000, Proteintech, 22018-1-AP), Vimentin (1:2000, Proteintech, 10366-1-AP), MMP2 (1:1000, Abcam, ab92536), MMP9 (1:1000, Abcam, ab76003), GM130 (1:1000, Abcam, ab52649), GRP94 (1:1000, Abcam, ab238126), ALIX (1:10,000, Abcam, ab186429), TSG101 (1:1000, Abcam, ab125011), CD63 (1:1000, Abcam, ab134045), CD81 (1:1000, Abcam, ab109201), ENO1 (1:1000, Abcam, ab155102), HA (1:4000, Abcam, ab9110), integrin α1 (1:500, Proteintech, 22146-1-AP), integrin α2 (1:10,000, Abcam, ab133557), integrin α3 (1:500, Proteintech, 66070-1-Ig), integrin α5 (1:1000, Abcam, ab150361), integrin α6 (1:2000, Abcam, ab181551), integrin αV (1:5,000, Abcam, ab179475), integrin β1 (1:1000, Abcam, ab52971), integrin β3 (1:1000, Abcam, ab119992), integrin β4 (1:1000, Abcam, ab182120), integrin β6 (1:10,000, Abcam, ab187155), FAK (1:2000, Abcam, ab40794), FAK (Y397) (1:1000, Abcam, ab81298), FAK (Y576 + Y577) (1:50,000, Abcam, ab76244), FAK (Y861) (1:10,000, Abcam, ab81293), FAK (Y925) (1:1000, Abcam, ab230813), Src (1:10,000, Abcam, ab109381), Src (Y418) (1:1000, Abcam, ab40660), Src (Y419) (1:5000, Abcam, ab185617), Src (Y529) (1:5000, Abcam, ab32078), p38MAPK (1:1000, Abcam, ab170099), p38MAPK (T180 + Y182) (1:1000, Abcam, ab195049), GAPDH (1:10,000, Abbkine, A01020, USA), and β-tubulin (1:10,000, Abbkine, A01030) were diluted in primary antibody diluent (KeyGEN BioTECH) and incubated with membranes overnight at 4 °C. The next day, the membranes were washed three times with TBS-T and were then incubated with secondary antibody (1:10,000, Abbkine, A21020) for 1 h at room temperature. Membranes were washed three times with TBS-T before scanning and visualization of immunoreactions using the ECL western blot substrate (NCM Biotech, China). Protein expression of three independent experiments was quantified using ImageJ software.

### Exosome isolation, characterization, and analyses

Exosomes were isolated for mass spectrometry using an Exosome Isolation Kit (PTM BIO, China). Exosomes used for all other experiments were isolated by ultracentrifugation. The indicated cells at 70–80% confluence were cultured in medium supplemented with 10% exosome-depleted FBS for 72 h. Supernatant fractions collected from 72-h cell cultures were centrifuged at 300 × g for 10 min, 2000 × *g* for 20 min, and 10,000 × *g* for 30 min. The supernatant fractions were then filtered through a 0.22-µm filter. Exosomes were collected by ultracentrifugation at 100,000 × *g* for 70 min at 4 °C. The exosome pellet was resuspended in 10 ml of PBS and purified by ultracentrifugation at 100,000 × *g* for 70 min at 4 °C (Beckman, USA). The purified exosomes were resuspended in PBS for subsequent experiments. The exosomal protein concentration was measured by using a BCA Protein Assay Kit. Exosome preparation was verified by western blot analysis and electron microscopy. Purified exosomes were dropped onto electron microscopy grids, allowed to absorb for 10 min, and then negatively stained with 2% phosphotungstic acid for 5 min. The electron microscopy grids were observed under a transmission electron microscope (JEOL, Japan) at 120 kV. Exosome sizes and particle numbers were assessed using a Zeta View PMX110 (Particle Metrix, Germany) equipped with a blue laser (405 nm) and analyzed using nanoparticle tracking analysis (NTA) software (Zeta View 8.02.28).

### Exosome labeling and tracing

Purified exosomes were fluorescently labeled using a PKH67 Green Fluorescent Cell Linker Kit (Sigma) according to the manufacturer’s protocol. Labeled exosomes were washed in 10 ml of PBS and recollected by ultracentrifugation. Purified exosomes labeled with PKH67 were resuspended in serum-free medium and then used for culture with cells for 2 h. Subsequently, cells were fixed in 4% paraformaldehyde for 30 min, and cell nuclei were counterstained with DAPI for 5 min at room temperature in the dark. Fluorescence signals were visualized using a Leica TCS SP5II confocal microscope.

### Animal study

The 3- to 4-week-old female BALB/c-nu/nu nude mice were randomized. Approximately 1 × 10^6^ cells were suspended in 30 μl of PBS/Matrigel (1:2) cocktail or 30 μl of exosome/Matrigel (1:2) cocktail containing 5 μg of exosomes. Through a 1-cm transverse incision in the upper abdomen, they were orthotopically injected in the hepatic lobe of each nude mouse anesthetized with pentobarbital sodium. Another approximately 1 × 10^6^ cells were suspended in 100 μl of PBS or 100 μl of exosomes (50 μg/ml) and were injected intravenously into each nude mouse. After 4 or 8 weeks, the nude mice were euthanized, and their livers and lungs were harvested and analyzed to assess HCC cell proliferation and metastasis in vivo. All nude mice were purchased by and raised in the Experimental Animal Center of Dalian Medical University. All nude mice were raised in a specific pathogen-free (SPF) environment and were provided sterilized feed and water. All animal experiments were approved by the Experimental Animal Ethics Committee of Dalian Medical University. All animals received humane care according to the criteria outlined in the Guide for the Care and Use of Laboratory Animals prepared by the National Academy of Sciences and published by the National Institutes of Health.

### Establishment of stable overexpression and knockdown cell lines

For knockdown of ENO1 in HCC cell lines, shRNA sequences targeting ENO1 (shENO1-1: 5′-GGACTTCAAGTCTCCCGATGA-3′, shENO1-2: 5′-GCGGTTCTCATGCTGGCAACA-3′, shENO1-3: 5′-GCTCAAAGTCAACCAGATTGG-3′) were inserted into the LV3 lentiviral vector and were then packaged in 293T cells using RNAi-Mate (GenePharma, China). The shENO1 vector (LV3-shENO1) or mock vector (LV3-NC) was transfected into HCCLM3 cells. For overexpression of ENO1 in HCC cell lines, the full-length cDNA encoding ENO1 was inserted into the LV5 lentiviral vector and was then packaged in 293T cells using RNAi-Mate. The ENO1 vector (LV5-ENO1) or mock vector (LV5-NC) was transfected into MHCC97L and HepG2 cells. Transfected cells used for overexpression or knockdown were selected with puromycin (5 μg/ml) for 2 weeks. The efficiency of stable overexpression or knockdown was evaluated by western blotting.

### Proteomics analysis

The exosome pellet was washed with PBS and sonicated three times on ice using a high-intensity ultrasonic processor (Scientz, China) in 4 volumes of lysis buffer (8 M urea, 1% protease inhibitor cocktail). The remaining debris was removed by centrifugation at 12,000 × *g* for 10 min at 4 °C. Finally, the supernatant was collected, and the protein concentration was measured with a BCA protein assay kit according to the manufacturer’s instructions. For digestion, the proteins in solution were reduced with 5 mM dithiothreitol for 30 min at 56 °C and alkylated with 11 mM iodoacetamide for 15 min at room temperature in the dark. The protein sample was then diluted by adding 100 mM tetraethylammonium bromide (TEAB) to a urea concentration of less than 2 M. Finally, trypsin was added at a 1:50 trypsin:protein mass ratio for the first digestion overnight and a 1:100 trypsin:protein mass ratio for a second digestion for 4 h. After trypsin digestion, peptides were desalted on a Strata X C18 SPE column (Phenomenex) and dried in a vacuum. Peptides were reconstituted in 0.5 M TEAB and processed according to the manufacturer’s protocol for the tandem mass tag (TMT) kit. Tryptic peptides were fractionated via high-pH reverse-phase HPLC using an Agilent 300Extend-C18 column (5-μm particles, 4.6-mm inner diameter (ID), 250-mm length). Tryptic peptides were dissolved in solvent A (0.1% formic acid) and loaded directly onto an in-house-constructed reversed-phase analytical column (15-cm length, 75-μm ID). The gradient was composed of an increase from 6 to 20% solvent B (0.1% formic acid in 90% acetonitrile) over 20 min, from 20 to 35% solvent B over 13 min, and from 35 to 80% over 3 min, followed by a final holding step at 80% for 3 min. All steps were performed at a constant flow rate of 300 nL/min on an EASY-nLC 1000 UPLC system. Peptides were subjected to nanospray ionization (NSI) tandem mass spectrometry (MS/MS) in a Q Exactive^TM^ Plus (Thermo Fisher Scientific) coupled online to the UPLC system. The applied electrospray voltage was 2.0 kV. The m/z scan range was 400–1500 for a full scan, and intact peptides were detected in the Orbitrap at a resolution of 70,000. Peptides were then selected for MS/MS using a normalized collision energy (NCE) setting of 100, and fragments were detected in the Orbitrap at a resolution of 17,500. A data-dependent procedure alternating between one MS scan and 20 subsequent MS/MS scans with a dynamic exclusion window of 15.0 s was used. The automatic gain control (AGC) was set at 5 × 10^4^. The resulting MS/MS data were processed using the MaxQuant search engine (v.1.5.2.8). Tandem mass spectra were searched against the SwissProt Human database concatenated with the reverse decoy database. Trypsin/P was specified as the cleavage enzyme, allowing up to 2 missed cleavages. The mass tolerance for precursor ions was set at 20 ppm in the first search and 5 ppm in the main search, and the mass tolerance for fragment ions was set at 0.02 Da. The false discovery rate (FDR) was adjusted to 1%.

### Immunohistochemistry (IHC) staining and scoring

After deparaffinization with xylene, rehydration in an ethanol gradient, heat-mediated antigen retrieval in citric acid and immersion in 3% hydrogen peroxide, tissue sections were incubated with the antibody against ENO1 (1:100, Abcam, ab155955) overnight at 4 °C. The next day, tissue sections were incubated with an HRP-conjugated secondary antibody (1:1000, Abcam, ab6721) for 1 h at room temperature. Tissue sections were developed with DAB solution and examined under a microscope. Tissue sections were counterstained with hematoxylin. The ENO1 expression score was assessed according to both the proportion (negative, scored 0; 1–25%, scored 1; 25–50%, scored 2; 50–75%, scored 3; and 75–100%, scored 4) and intensity (negative, scored 0; 1+, scored 1; 2+, scored 2; 3+, scored 3) of positively stained cells. Finally, the product of the “staining intensity score” and “positive staining rate score” was used as the total score for grouping. According to the data distribution, sections with a total score of 4 or less were considered the low ENO1 expression group, and sections with a total score of greater than 4 were considered the high ENO1 expression group. These scores were determined independently by two experienced pathologists in a blinded manner, and the mean percentage values were used.

### Statistical analysis

Data are expressed as the mean ± standard deviations (SD) and analyzed by GraphPad Prism software. The data of different experimental groups meet normal distribution and the variance is similar between the groups. Statistical significance was analyzed by two-tailed Student’s *t*-test or ANOVA where applicable. The correlation of ENO1 expression with clinicopathological parameters in HCC patients was evaluated by the chi-square test and Fisher’s exact probability method. Survival curves were constructed via the Kaplan–Meier method and analyzed by the log-rank test. Univariate and multivariate analyses based on the Cox proportional hazards regression model were performed to identify the factors with a significant influence on survival. *P* < 0.05 was considered statistically significant.

## Results

### Exosomes derived from highly metastatic HCC cells promote malignant transformation of HCC cells with low metastatic potential in vitro

**Fig. 1:**
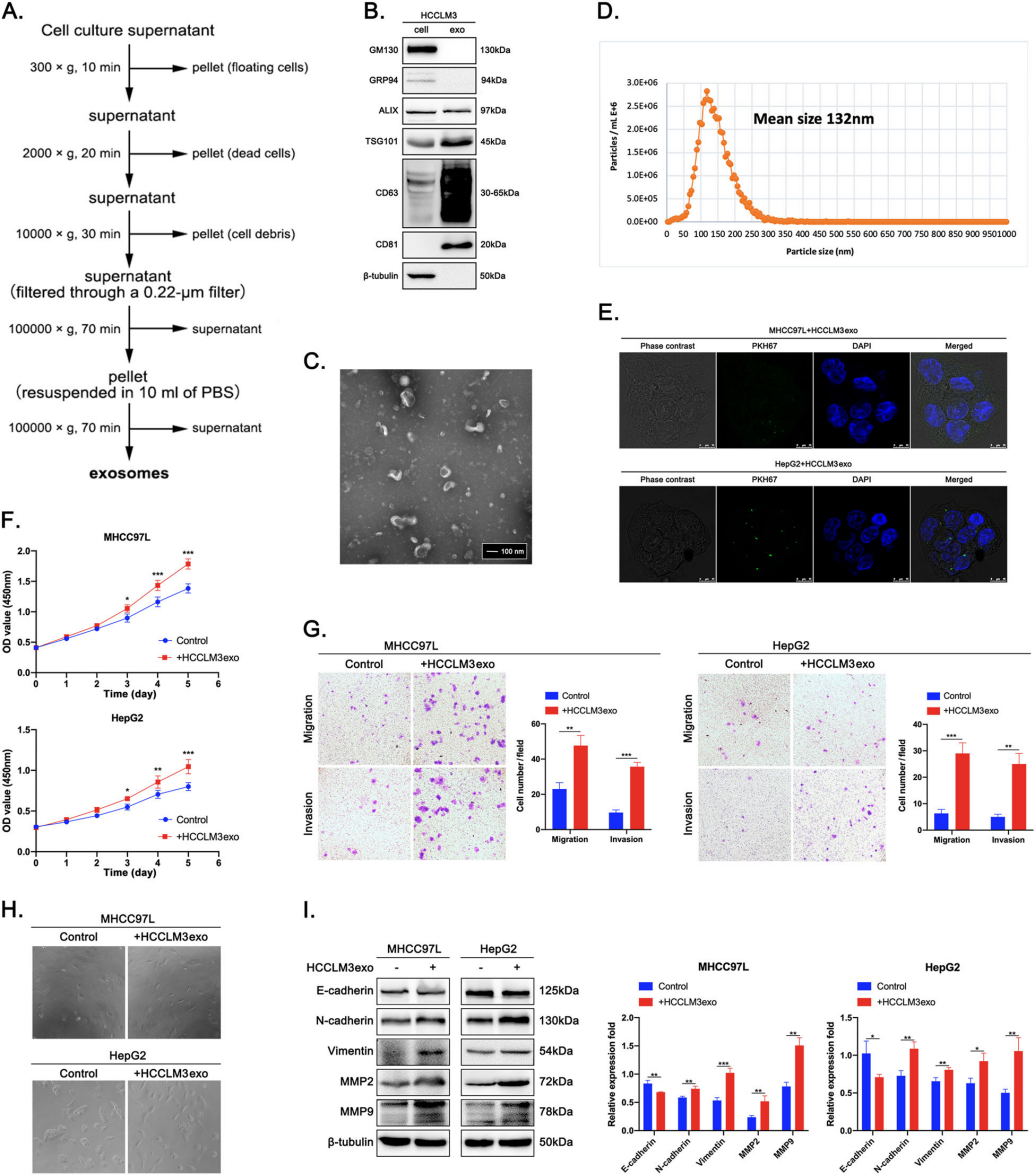
HCCLM3exos promote the malignant transformation of MHCC97L and HepG2 cells in vitro. **A** Flow chart for the exosome purification procedure based on differential ultracentrifugation. **B** Western blot analysis of exosomal positive protein marker (ALIX, TSG101, CD63, and CD81) and negative protein marker (GM130, GRP94) in HCCLM3 cells and in HCCLM3 cell-derived exosomes. **C** A representative electron microscope image of HCCLM3 cell-derived exosomes. Scale bar represent 100 nm. **D** Size, number and distribution analysis of HCCLM3 cell-derived exosomes by NTA. **E** Fluorescence microscopy analysis of PKH67-labeled exosome incorporation (green) by MHCC97L and HepG2 cells. Exosomes were isolated from HCCLM3 cells. Scale bar represent 10 μm. **F** CCK-8 assays, **G** Transwell migration and invasion assays, **H** Morphological analysis of MHCC97L and HepG2 cells educated with HCCLM3 cell-derived exosomes (50 μg/ml). **I** Western blot analysis of EMT-related protein levels (E-cadherin, N-cadherin, Vimentin, MMP2, and MMP9) in MHCC97L and HepG2 cells educated with HCCLM3 cell-derived exosomes (50 μg/ml). A representative of three independent experiments is shown. All data are represented as mean ± SD. *P < 0.05, **P < 0.01, ***P < 0.001 according to two-tailed Student’s *t*-test.

Studies have shown that metastasis-related subclones or specific mutations usually occur during the progression of malignant tumors. Not only are there differences between different tumors, but there are also tumor cells with different malignant phenotypes in the same tumor^33,34^. To demonstrate the effect of highly metastatic HCC cell-derived exosome education, we first evaluated the malignant phenotypes of three HCC cell lines with different metastatic potential constructed based on the same parental cell line by the Liver Cancer Institute & Zhongshan Hospital of Fudan University^35–38^. CCK-8 and colony formation assays showed that HCCLM3 cells had significantly higher proliferative activity than MHCC97L and MHCC97H cells (Supplementary Fig. 1A, B). Wound healing and Transwell assays showed that HCCLM3 cells had significantly greater migratory and invasive capacities than MHCC97L and MHCC97H cells (Supplementary Fig. 1C, D). Epithelial-mesenchymal transition (EMT) is an important biological process during the development of malignant tumors. Through EMT, tumor cells lose their polarity and connection to the basement membrane, and they acquire mesenchymal features such as increased migratory and invasive abilities, apoptosis resistance, and extracellular matrix degradation, leading to tumor recurrence and metastasis^39^. IF staining and western blot results revealed significant activation of EMT in HCCLM3 cells compared to MHCC97L and MHCC97H cells (Supplementary Fig. 1E, F). Subsequently, highly metastatic HCCLM3 cell-derived exosomes (HCCLM3exos) were isolated by differential ultracentrifugation (Fig. 1A) and verified by detecting the expression of positive protein markers (ALIX, TSG101, CD63, CD81) and negative protein markers (GM130, GRP94) (Fig. 1B). Under electron microscopy, the purified exosomes exhibited a typical cup-shaped morphology (Fig. 1C). NTA indicated that the particle number of exosomes purified from cultures of 3 × 10^8^ cells was 1.2E + 12 particles/ml, and most of the particles were within the size range of exosomes, with an average diameter of 132 nm (Fig. 1D).

Two cell lines with relatively low metastatic potential, MHCC97L, and HepG2, were used to further explore the effect of HCCLM3exo education. MHCC97L and HepG2 cells were educated with PKH67-labeled HCCLM3exos, and confocal microscopy imaging showed that uptake of HCCLM3exos not only occurred in MHCC97L cells from the same parental cell line, but also in HepG2 cells from a different parental cell line (Fig. 1E). CCK-8 and Transwell assays showed that the proliferative activity and migratory and invasive capacities of MHCC97L and HepG2 cells were significantly enhanced after HCCLM3exo education compared to treatment with PBS (Fig. 1F, G). Under the microscope, MHCC97L, and HepG2 cells changed from oval shaped to spindle shaped and lost their cellular connections after HCCLM3exo education (Fig. 1H). E-cadherin was significantly downregulated, but N-cadherin, Vimentin, MMP2, and MMP9 were significantly upregulated in MHCC97L and HepG2 cells after HCCLM3exo education, indicating that HCCLM3exo-educated MHCC97L and HepG2 cells undergo EMT (Fig. 1I). Taken together, these data show that exosomes derived from highly metastatic HCC cells can act as tumor promoters and instigate malignant transformation of HCC cells with low metastatic potential.

### Exosomes derived from highly metastatic HCC cells promote the growth and metastasis of HCC cells with low metastatic potential in vivo

**Fig. 2:**
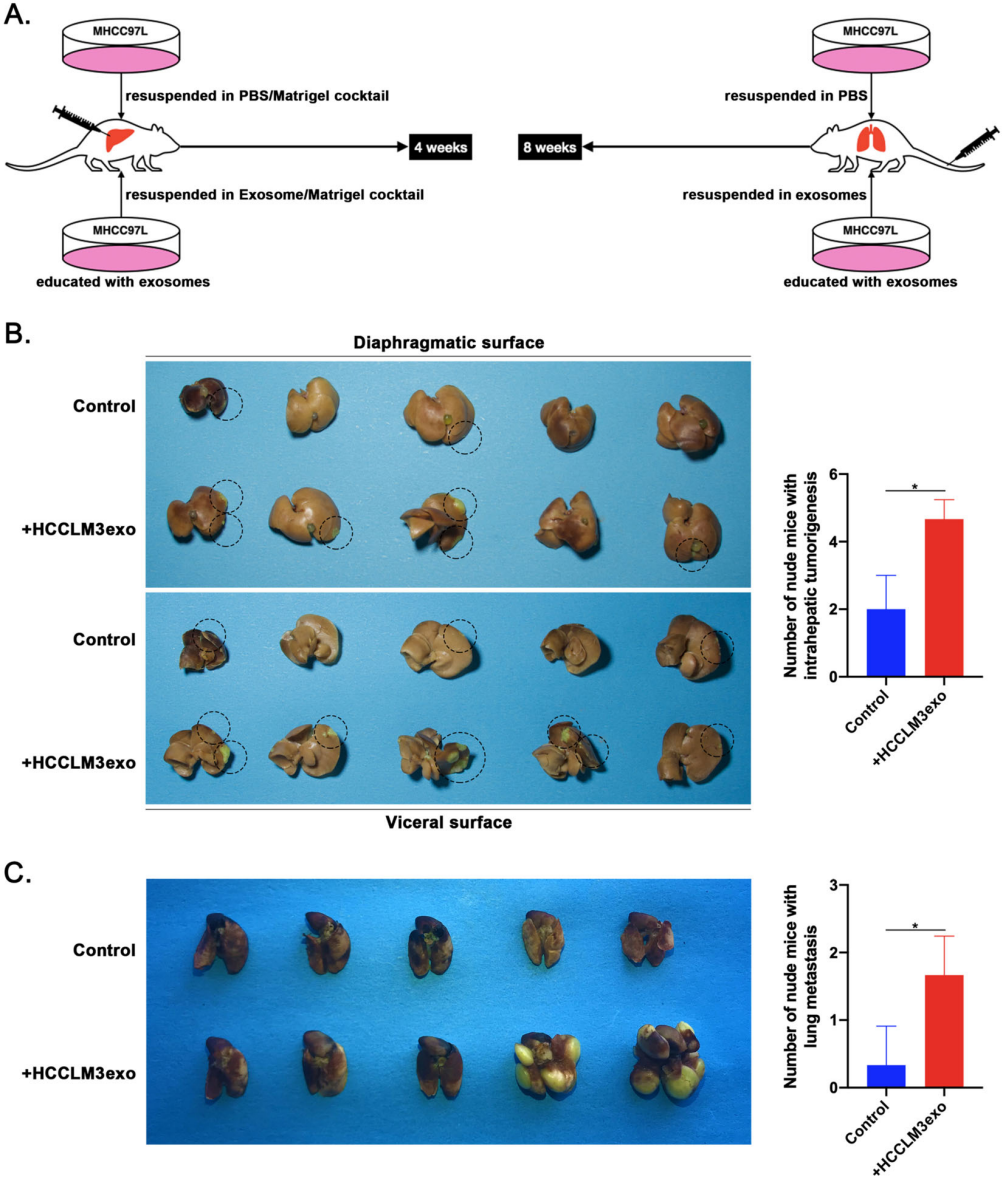
HCCLM3exos promote the growth and metastasis of MHCC97L cells in vivo. **A** A flow chart depicting the in vivo experimental design. **B** Evaluation of HCC growth by tumor size and number of nude mice with intrahepatic tumorigenesis in mice injected orthotopically with MHCC97L cells following education with PBS or HCCLM3exos. **C** Evaluation of HCC metastasis by tumor size and number of nude mice with lung metastasis in mice injected in the tail vein with MHCC97L cells following education with PBS or HCCLM3exos. For the in vivo experiments, five nude mice per group were used. A representative of three independent experiments is shown. All data are represented as mean ± SD. *P < 0.05 according to two-tailed Student’s *t*-test.

To determine whether HCCLM3exo plays a vital role in HCC progression in vivo, we used in vivo experimental models of intrahepatic injection and tail vein injection. Tumorigenic MHCC97L cells with low metastatic potential used in the experimental group were educated for 24 h in medium supplemented with HCCLM3exos (50 μg/ml) prior to injection, while the control cells were not educated. The MHCC97L cells of the two groups were subjected to routine digestion and centrifugation. After washing with PBS, cells were resuspended in exosome/Matrigel cocktail containing 5 μg of HCCLM3exos and PBS/Matrigel cocktail, respectively, and then orthotopically injected into the hepatic lobe of nude mice and cultured for 4 weeks. For the tail vein injection model, two groups of MHCC97L cells treated under the same conditions were resuspended in 100 μl of PBS or 100 μl of HCCLM3exos (50 μg/ml) and then intravenously injected into nude mice and cultured for 8 weeks (Fig. 2A). As expected, intrahepatic tumorigenesis and lung metastases of MHCC97L cells were frequently detected in the exosome-educated group but rarely in the non-exosome-educated group. The growth rate of MHCC97L cells in the liver and lung was increased significantly after HCCLM3exo education, and some MHCC97L cells in the exosome-educated group developed intrahepatic metastasis (Fig. 2B, C). Therefore, we speculate that certain specific proteins in highly metastatic HCC cells may be transferred to other HCC cells via exosome-mediated crosstalk, thereby promoting HCC growth and metastasis.

### ENO1 expression is significantly upregulated in highly metastatic HCC cells and their exosomes and is closely associated with clinicopathological features

**Fig. 3:**
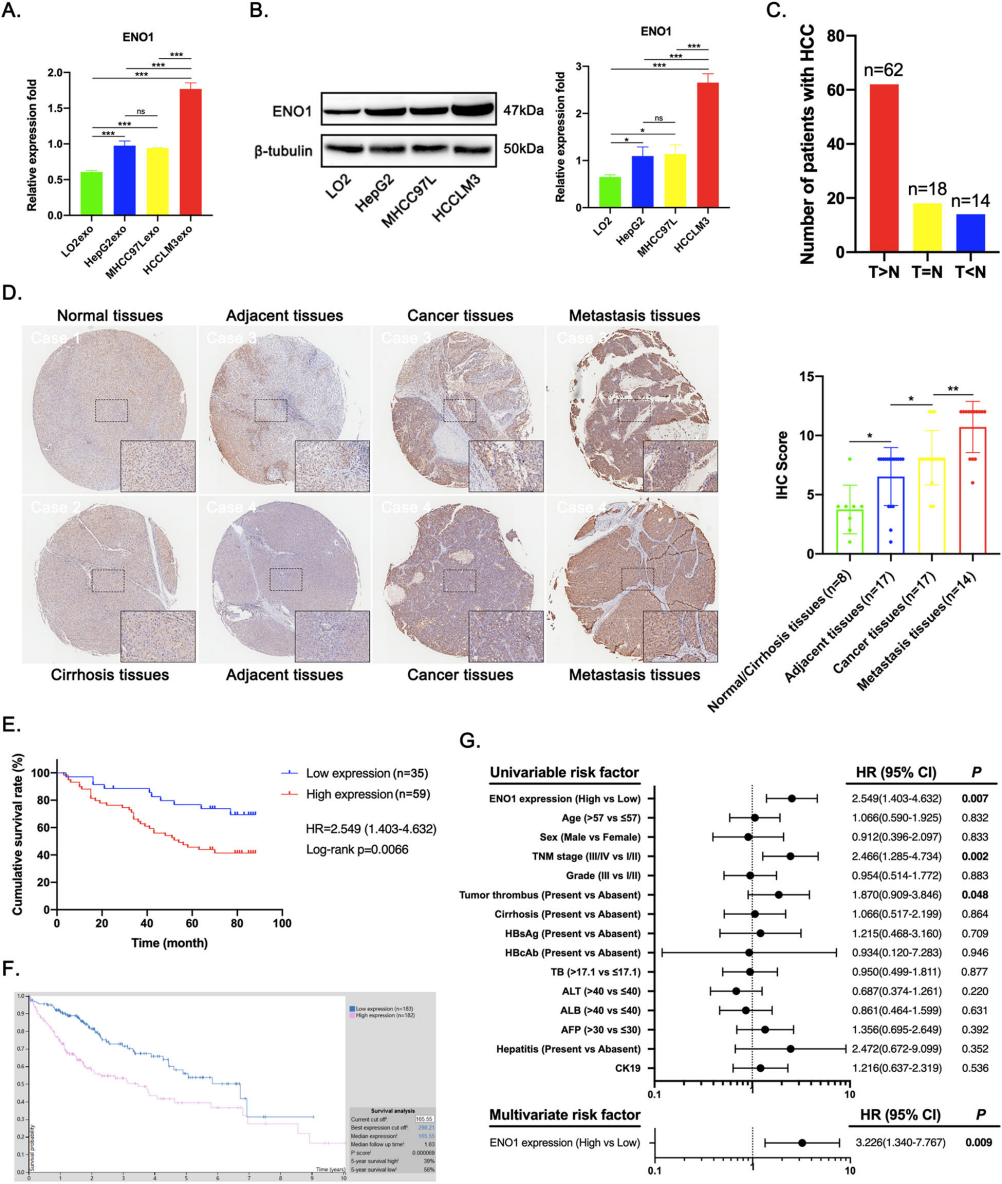
Increased ENO1 expression is correlated with poor prognosis of HCC patients. **A** The expression level of ENO1 in LO2exo, HepG2exo, MHCC97Lexo, and HCCLM3exo determined by proteomics analysis. **B** Western blot analysis of ENO1 levels in LO2, HepG2, MHCC97L, and HCCLM3 cells. A representative of three independent experiments is shown. **C** Differential expression of ENO1 in 94 pairs of HCC and adjacent non-tumor tissues. **D** IHC staining of ENO1 expression in normal liver tissues, cirrhosis tissues, paired of HCC and non-tumor tissues and metastasis tissues. Representative photographs of ENO1 staining in different tissues are shown. Scale bar represent 800 μm and 100 μm. **E** Kaplan–Meier analysis of overall survival of 94 HCC patients with different ENO1 expression levels. **F** Kaplan–Meier analysis of overall survival of 365 HCC patients in the TCGA cohort. **G** Forest plot showing the association between ENO1 expression and HCC survival as revealed by univariate and multivariate analyses (HR, hazard ratio; CI, confidence interval). All data are represented as mean ± SD. *P < 0.05, **P < 0.01, ***P < 0.001 according to two-tailed Student’s *t*-test.

To clarify the exosomal protein expression profiles of hepatocytes and HCC cells with different metastatic potentials, we first performed TMT quantitative proteomic analysis of exosomes isolated from cell supernatants. A total of 19,497 peptides, including 18,764 specific peptides, and 1613 proteins, including 1389 quantifiable proteins, were identified (Supplementary Fig. 2A). We obtained the quantitative values for each sample in triplicate. For differences with *P* < 0.05, a difference in the expression level of greater than 1.5 was considered the threshold for a significant increase, and a difference in the expression level of less than 0.667 was considered the threshold for a significant decrease. Comparison between the groups revealed approximately 200 differentially expressed proteins that were significantly upregulated or downregulated (Supplementary Fig. 2B). Interestingly, we combined with the results of Kaplan–Meier analysis of overall survival found that a greater variety of proteins related to poor prognosis of HCC patients were highly enriched in HCC cell-derived exosomes to varying degrees, and there are more unhealthy proteins in HCCLM3exo, suggesting that these proteins may be the key to promoting HCC progression via exosome-mediated crosstalk (Supplementary Fig. 2C). To gain an in-depth understanding of the specific mechanisms by which exosomes derived from highly metastatic HCC cells promote the malignant transformation of HCC cells with low metastatic potential and promote HCC growth and metastasis, we further analyzed exosomal MS data and found that the expression of ENO1, a potential promoter of HCC progression, in addition to being highly enriched in HCCLM3exos, was also significantly upregulated in MHCC97Lexos and HepG2exos compared to in LO2exos. Although it cannot fully reflect the proliferative activity of cells, the expression level of exosomal ENO1 was positively correlated with cell migration and invasion capabilities (Supplementary Fig. 3). To verify the hypothesis that the differential expression of exosomal ENO1 is due to differences at the cellular level, we assessed ENO1 expression in cells. The western blot results showed that the expression trend of ENO1 in cells was consistent with that in exosomes, with significantly increased expression in highly metastatic HCCLM3 cells (Fig. 3A, B).

To observe the role of ENO1 in HCC progression, 94 pairs of HCC and adjacent non-tumor tissues were examined for ENO1 expression. As a result, ENO1 expression was upregulated in 62 out of 94 HCC cases (Fig. 3C). In addition, IHC staining of a tissue microarray containing 2 normal liver tissues, 6 cirrhosis tissues, 17 paired of HCC and non-tumorous tissues and 14 HCC metastasis tissues showed that ENO1 expression was significantly higher in HCC metastasis tissues than in the other tissue types assessed (Fig. 3D). Subsequently, we analyzed the correlation between ENO1 expression and the clinicopathological characteristics of HCC patients. As shown in Table 1, ENO1 expression was significantly associated with the tumor-node-metastasis (TNM) stage and differentiation grade. High ENO1 expression was significantly associated with poor overall survival (Fig. 3E). The survival analysis of The Cancer Genome Atlas (TCGA) cohort showed the same trend (Fig. 3F). Univariate and multivariate Cox regression analyses further identified ENO1 expression as an independent predictor of the survival of HCC patients (Fig. 3G). The above results indicate that high ENO1 expression, as a risk factor for metastasis, is significantly correlated with poor prognosis in HCC, so ENO1 may play an important role in the malignant progression of HCC.

**Table 1:** Correlations between ENO1 expression and clinicopathological parameters in patients with HCC.

Clinicopathological parameter	Variables	ENO1 expression		Total	*χ*^2^	*P* value^b^
		Low	High			
Age (year)					1.138^a^	0.286
	≤57	20	27	47		
	>57	15	32	47		
Sex					0.044	0.833
	male	31	50	81		
	female	4	9	13		
TNM stage					4.932^a^	**0.026**
	I/II	27	32	59		
	III/IV	8	27	35		
Grade					5.894^a^	**0.015**
	I/II	27	32	59		
	III	7	27	34		
		1		1		
Tumor thrombus					2.956^a^	0.086
	−	24	35	59		
	+	5	19	24		
		6	5	11		
Cirrhosis					1.098^a^	0.295
	−	5	15	20		
	+	26	43	69		
		4	1	5		
HBsAg					0.013	0.908
	−	3	7	10		
	+	30	50	80		
		2	2	4		
HBcAb						0.527
	−	0	2	2		
	+	29	47	76		
		6	10	16		
TB					0.204^a^	0.652
	≤17.1	20	37	57		
	>17.1	12	18	30		
		3	4	7		
ALT					0.618^a^	0.432
	≤40	15	30	45		
	>40	19	27	46		
		1	2	3		
ALB					0.265^a^	0.607
	≤40	12	24	36		
	>40	19	30	49		
		4	5	9		
AFP					1.165^a^	0.28
	≤30	12	16	28		
	>30	17	38	55		
		6	5	11		
Hepatitis					0.118	0.731
	−	1	4	5		
	+	34	55	89		
CD34						0.526
	-	0	2	2		
	+	23	37	60		
		12	20	32		
CK19					0.144^a^	0.704
	−	23	41	64		
	+	12	18	30		
Recurrence or						
metastasis						0.725
	−	13	15	28		
	+	4	7	11		
		18	37	55		

^a^0 cells (0.0%) have expected count less than 5. ^b^*P* value was calculated by χ^2^ test of Fisher’s exact test.

The bold number represents the *p*-values with significant differences.

### ENO1 promotes the proliferation, migration, invasion, and EMT of HCC cells in vitro

**Fig. 4:**
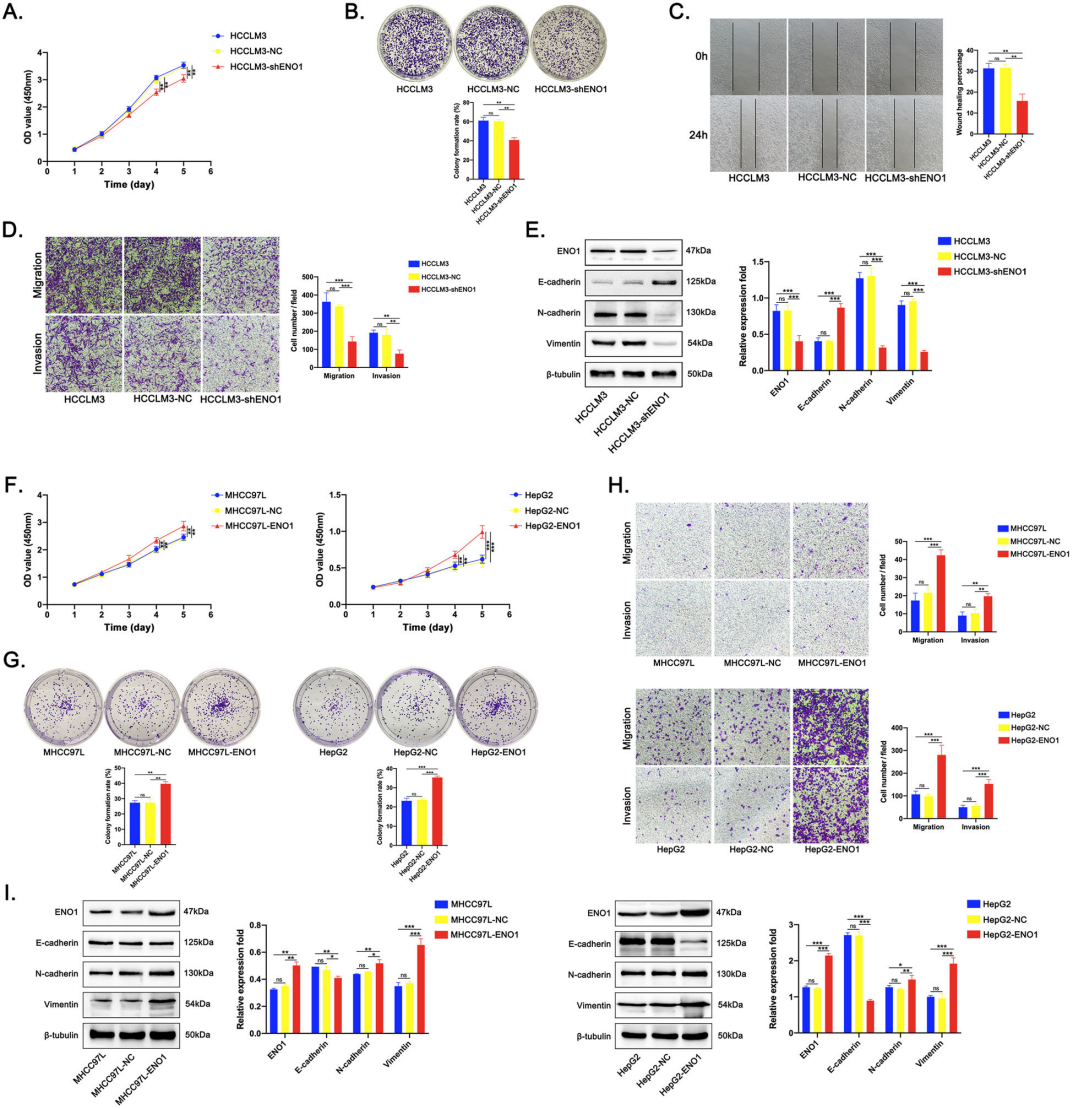
ENO1 promotes HCC cell proliferation, migration and invasion and EMT in vitro. **A** CCK-8 assays, **B** colony formation assays, **C** wound healing assays, **D** transwell migration and invasion assays of HCCLM3 cells after ENO1 knockdown. **E** Western blot analysis of E-cadherin, N-cadherin and Vimentin levels in HCCLM3 cells after ENO1 knockdown. **f** CCK-8 assays, **G** colony formation assays, **H** transwell migration and invasion assays of MHCC97L and HepG2 cells after ENO1 overexpression. **I** Western blot analysis of E-cadherin, N-cadherin and Vimentin levels in MHCC97L and HepG2 cells after ENO1 overexpression. A representative of three independent experiments is shown. All data are represented as mean ± SD. *P < 0.05, **P < 0.01, ***P < 0.001 according to two-tailed Student’s *t*-test.

We have confirmed that higher level of ENO1 proteins was in HCCLM3 cells, but low levels were in MHCC97L and HepG2 cells (Fig. 3B). To further explore the function of endogenous ENO1 in HCC cells, we used lentivirus technology to induce stable ENO1 knockdown in HCCLM3 cells and ENO1 overexpression in MHCC97L and HepG2 cells, which were used for loss-of-function and gain-of-function studies (Supplementary Fig. 5A, B). CCK-8 and colony formation assays showed that ENO1 knockdown significantly inhibited proliferative activity in HCCLM3 cells (Fig. 4A, B). Wound healing and Transwell assays showed that ENO1 knockdown significantly reduced migration and invasion in HCCLM3 cells (Fig. 4C, D). Moreover, the western blot results revealed that ENO1 knockdown upregulated E-cadherin expression, but downregulated N-cadherin and Vimentin expression in HCCLM3 cells (Fig. 4E). In contrast, ENO1 overexpression significantly promoted proliferative activity in MHCC97L and HepG2 cells (Fig. 4F, G). Transwell assays showed that ENO1 overexpression significantly enhanced migration and invasion in MHCC97L and HepG2 cells (Fig. 4H). The western blot results revealed that ENO1 overexpression downregulated E-cadherin expression, but upregulated N-cadherin and Vimentin expression in MHCC97L and HepG2 cells (Fig. 4I). Taken together, these data indicate that increased ENO1 expression promotes the proliferation, migration, invasion, and EMT of HCC cells.

### ENO1 is transferred between HCC cells via exosomes to promote HCC growth and metastasis

**Fig. 5:**
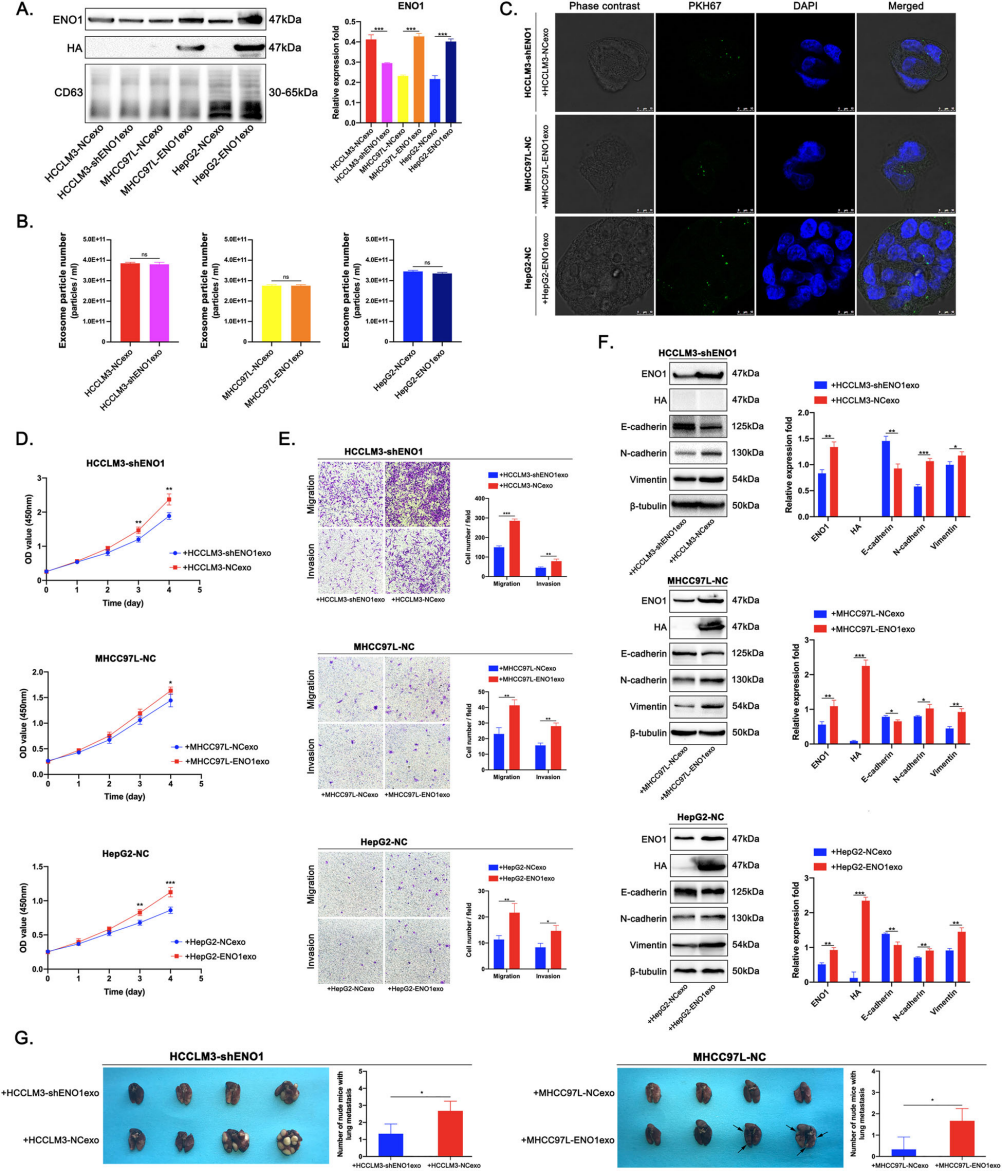
Exosome-derived ENO1 promotes HCC growth and metastasis. **A** Western blot analysis of ENO1 level in exosomes derived from HCCLM3-NC, HCCLM3-shENO1, MHCC97L-NC, MHCC97L-ENO1, HepG2-NC, and HepG2-ENO1 cells, and HA protein expression in MHCC97L-ENO1exos and HepG2-ENO1exos. **B** NTA analysis of the impact of altered ENO1 expression in HCC cells on the number of exosomes released. **C** Fluorescence microscopy analysis of PKH67-labeled exosomes with high ENO1 expression incorporation (green) by HCCLM3-shENO1, MHCC97L-NC, and HepG2-NC cells with relatively low ENO1 expression. Exosomes were isolated from HCCLM3-NC, MHCC97L-ENO1 and HepG2-ENO1 cells. Scale bar represent 10 μm. **D** Cell CCK-8 assays, **E** transwell migration and invasion assays of HCCLM3-shENO1, MHCC97L-NC, and HepG2-NC cells educated with HCCLM3-NCexos, MHCC97L-ENO1exos, HepG2-ENO1exos or self-secreted exosomes (50 μg/ml). **F** Western blot analysis of ENO1, HA, E-cadherin, N-cadherin and Vimentin levels in HCCLM3-shENO1, MHCC97L-NC, and HepG2-NC cells educated with HCCLM3-NCexos, MHCC97L-ENO1exos, HepG2-ENO1exos or self-secreted exosomes (50 μg/ml). **G** Evaluation of HCC metastasis by tumor size and number of nude mice with lung metastasis in mice injected in the tail vein with HCCLM3-shENO1 and MHCC97L-NC cells following education with HCCLM3-NCexos, MHCC97L-ENO1exos or self-secreted exosomes. For the in vivo experiments, four nude mice per group were used. A representative of three independent experiments is shown. All data are represented as mean ± SD. *P < 0.05, **P < 0.01, ***P < 0.001 according to two-tailed Student’s *t*-test.

To confirm that exosome-derived ENO1 can be horizontally transferred between HCC cells, we used lentivirus technology to induce stable HA-tagged ENO1 overexpression in MHCC97L and HepG2 cells (Supplementary Fig. 5C). To further clarify whether ENO1 knockdown or overexpression in HCC cells can affect the expression level of ENO1 in their exosomes, we first measured exosomal ENO1 expression with or without ENO1 regulation. The western blot results showed that ENO1 expression was significantly reduced in exosomes derived from HCCLM3 cells with ENO1 knockdown, but was significantly increased in exosomes derived from MHCC97L and HepG2 cells with ENO1 overexpression compared to control exosomes. Moreover, we detected HA expression in exosomes derived from MHCC97L and HepG2 cells overexpressing ENO1 (Fig. 5A). NTA showed that there was no difference in the particle number of exosomes purified from cultures of 1 × 10^8^ cells with or without ENO1 regulation, indicating that ENO1 knockdown or overexpression in HCC cells did not affect the number of exosomes released (Fig. 5B).

By loss- and gain-of-function studies, we confirmed the role of intracellular ENO1 in HCC progression through the regulation of proliferation, migration, invasion and EMT (Fig. 4). We aimed to clarify whether exosome-derived ENO1 can also affect the abovementioned biological behaviors in recipient cells; we first confirmed the uptake of PKH67-labeled exosomes with high ENO1 expression in HCC cells with relatively low ENO1 expression via confocal microscopy (Fig. 5C). Subsequently, CCK-8 and Transwell assays showed that the proliferative activity and migratory and invasive capacities of HCCLM3-shENO1, MHCC97L-NC, and HepG2-NC cells were significantly enhanced after education with exosomes with high ENO1 expression compared to education with self-secreted exosomes (Fig. 5D, E). The western blot results showed that ENO1 expression was significantly increased in HCCLM3-shENO1, MHCC97L-NC, and HepG2-NC cells after education with exosomes with high ENO1 expression. Furthermore, HA protein was detected in MHCC97L-NC cells educated with MHCC97L-ENO1exos and HepG2-NC cells educated with HepG2-ENO1exos, indicating that exosome-derived ENO1 was horizontally transferred between HCC cells. In addition, we tested the effect of exosome-derived ENO1 on EMT in HCC cells with low ENO1 expression. Exosome-derived ENO1 decreased E-cadherin expression, but increased N-cadherin and Vimentin expression in HCCLM3-shENO1, MHCC97L-NC, and HepG2-NC cells (Fig. 5F). We further analyzed whether exosome-derived ENO1 may affect the lung metastasis of HCC in vivo. BALB/c-nu/nu mice were injected intravenously with tumorigenic HCCLM3-shENO1 and MHCC97L-NC cells educated with self-secreted exosomes or exosomes with high ENO1 expression. As shown in Fig. 5G, exosome-derived ENO1 significantly promoted the lung metastasis of HCCLM3-shENO1 and MHCC97L-NC cells. Therefore, these data indicate that exosome-derived ENO1, as a regulator of proliferation and metastasis transmissible between HCC cells, further promotes HCC growth and metastasis.

### Exosome-derived ENO1 upregulates integrin α6β4 expression and activates the integrin-mediated FAK/Src-p38MAPK pathway in HCC cells

**Fig. 6:**
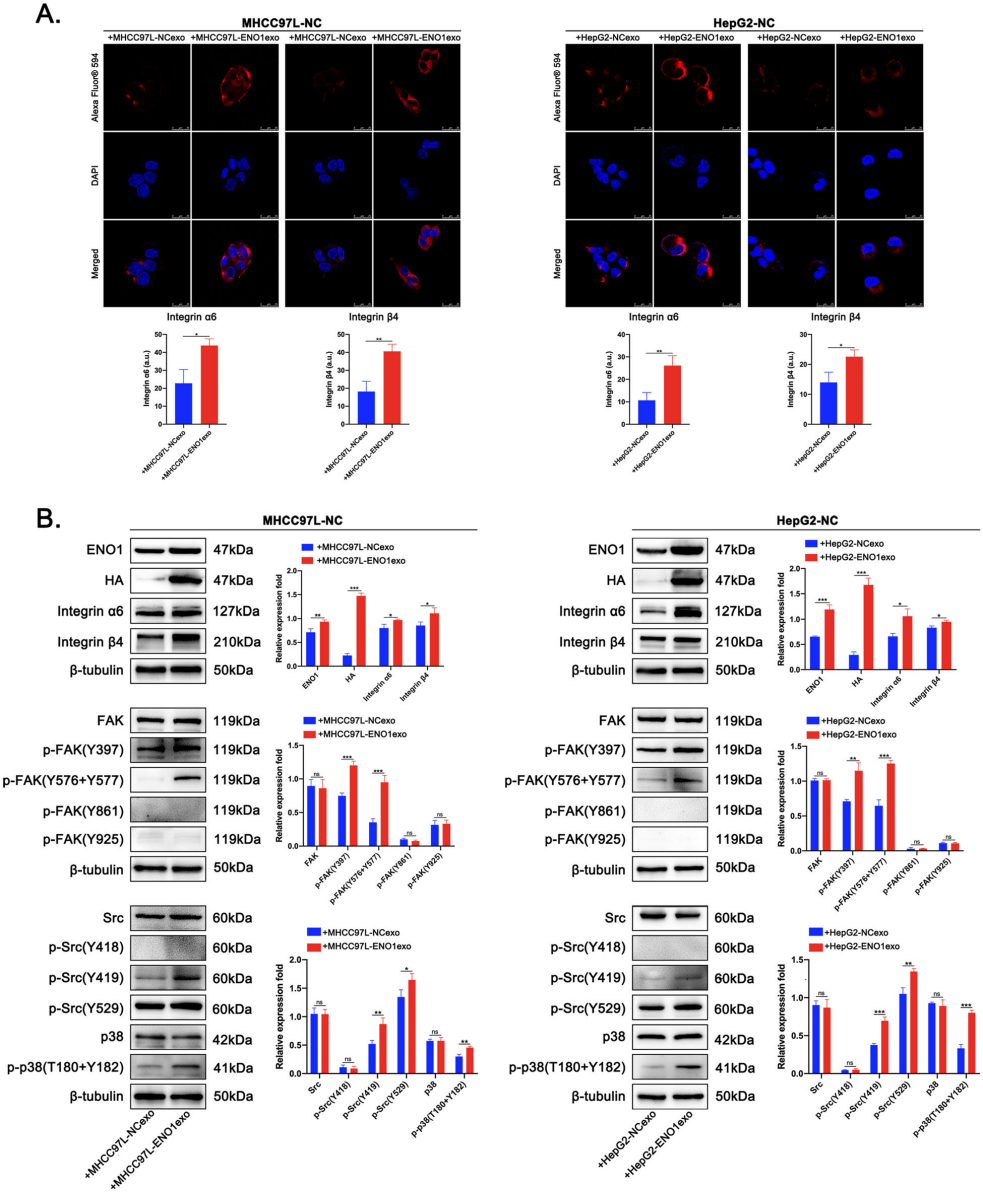
Exosome-derived ENO1 regulates integrin α6β4 expression and activates the FAK/Src-p38MAPK pathway in HCC cells. **A** Immunofluorescence quantification of integrin α6 and β4 expression in arbitrary units (a.u.) in MHCC97L-NC and HepG2-NC cells educated with MHCC97L-ENO1exos, HepG2-ENO1exos or self-secreted exosomes (50 μg/ml). Scale bar represent 25 μm. **B** Western blot analysis of the impact of exosome-derived ENO1 on the expression of integrin α6β4 and the activation of integrin-mediated FAK/Src-p38MAPK pathway in MHCC97L-NC and HepG2-NC cells educated with MHCC97L-ENO1exos, HepG2-ENO1exos or self-secreted exosomes (50 μg/ml). A representative of three independent experiments is shown. All data are represented as mean ± SD. *P < 0.05, **P < 0.01, ***P < 0.001 according to two-tailed Student’s *t*-test.

Studies have shown that, in addition to regulating the expression of metabolism-related proteins, ENO1 silencing also affects some adhesion and migration-related proteins, such as integrin family. The proteomics study by Capello et al. showed that ENO1 silencing can regulate some integrins expression in a human pancreatic cancer cell line, namely CFPAC-1 (Supplementary Fig. 6A)^24^. Therefore, we infer a possible role of integrins in regulating ENO1-mediated biological effects in HCC. Based on current reports on integrins in tumor research, especially in the HCC field^40–45^, we first tested the effect of ENO1 knockdown on the expression of related integrins (α1, α2, α3, α5, α6, αV, β1, β3, β4, and β6) in HCCLM3 cells. The results showed that ENO1 knockdown can downregulate integrin α1, α2, α6, and β4 but upregulate integrin αV expression in HCCLM3 cells. Among them, integrin α6 and β4 were significantly downregulated with ENO1 knockdown (Supplementary Fig. 6B). Second, the western blot results showed that ENO1 overexpression significantly upregulated integrin α6 and β4 expression in MHCC97L and HepG2 cells (Supplementary Fig. 6C), suggesting that ENO1 can regulate integrin α6β4 complexes in HCC cells to promote HCC progression. To further clarify whether exosome-derived ENO1 can also regulate integrin α6β4 expression in HCC cells with low ENO1 expression. By IF staining, we observed that the expression of integrin α6 and β4 was significantly increased in MHCC97L-NC and HepG2-NC cells after MHCC97L-ENO1exo and HepG2-ENO1exo education compared to control exosomes (Fig. 6A). Additional western blot results showed that the horizontal transfer of ENO1 with HA tags in MHCC97L-ENO1exos and HepG2-ENO1exos significantly upregulated integrin α6 and β4 expression in MHCC97L-NC and HepG2-NC cells compared to those treated with self-secreted exosomes (Fig. 6B). Exosome-derived ENO1 clearly led to excessive accumulation of integrin α6β4 in HCC cells with low ENO1 expression.

Integrins are the key players in tumor cell spread and are expressed at different levels at different stages of tumor development. Dysregulation of integrins can alter their functions, causing tumor cells to detach from the basement membrane and lose polarity, and these increased free integrins participate in bidirectional signal transduction in tumors^46–48^. Two key proteins, FAK and Src, important regulatory proteins affected by integrin α6β4, are closely related to tumor growth and metastasis and are activated through a series of phosphorylation events. To better clarify the effect of exosome-derived ENO1 on integrin α6β4-dependent signaling cascades, we evaluated the activation of the FAK/Src pathway and the downstream signaling molecule p38MAPK. The western blot results revealed that exosome-derived ENO1 promoted the phosphorylation of FAK at Tyr-397 and Tyr-576/577, the phosphorylation of Src at Tyr-419 and Tyr-529, and the phosphorylation of p38MAPK at Thr-180/182 in MHCC97L-NC and HepG2-NC cells (Fig. 6B). Collectively, these findings indicate that exosome-derived ENO1 facilitates the expression of integrin α6β4 and the activation of the FAK/Src pathway, supporting downstream signaling via p38MAPK in HCC cells.

## Discussion

The diversity of HCC cells is the key factor in the treatment failure and death of HCC patients. HCC cells have different degrees of heterogeneity both within the same HCC patient and among HCC patients, differentially remodeling microenvironments and ultimately determining the development direction of HCC. The daughter cells produced during the multiple divisions and proliferation of HCC cells exhibit different genetic and molecular biological alterations^33,49^. HCC cells with different degrees of malignancy exist at different stages of HCC development, and these cells can communicate with each other via exosome-mediated crosstalk mechanisms. Our study showed that exosomes derived from HCC cells with a higher degree of malignancy can educate HCC cells with a lower degree of malignancy and promote their malignant transformation. A similar phenomenon was supported by several studies showing that this effect of exosome education is not limited to tumor cells but also exists between tumor cells and stromal cells^50–52^. Numerous studies have confirmed that exosomes play important roles in the multistep process of HCC progression from chronic hepatitis B virus (HBV)/hepatitis C virus (HCV) infection to fibrosis/cirrhosis to early HCC to metastatic HCC^53–58^. Our data and the data of ExoCarta, an exosome database, consistently showed that the expression of proteins in HCC cell-derived exosomes is significantly different from that in hepatocyte-derived exosomes. Some proteins in HCC cell-derived exosomes can partially reflect the cellular characteristics and are significantly closely related to the poor prognosis of HCC. This close relationship not only indicates that these dysregulated proteins may determine the occurrence and developmental direction of HCC but also implies that the protein components in HCC-derived exosomes could become critical biomarkers for screening and prognosis assessment of HCC in the future.

ENO1 is localized to the cytoplasm and cell membrane but, as an important extracellular protein, can also be transferred between cells via exosomes. In the current study, we found that exosomal ENO1 levels depended on changes at the cellular level. ENO1 expression was significantly increased in HCC cell-derived exosomes compared to hepatocyte-derived exosomes. Moreover, as the malignancy of HCC cells increased, the ENO1 expression level increased correspondingly. Clinical data showed that ENO1 expression in metastatic lesions was higher than that in the primary lesions and that ENO1 upregulation was significantly correlated with the TNM stage and tumor differentiation grade, and predicted a poor prognosis in patients with HCC. These data correlated well with previous observations in lung cancer^25^, pancreatic cancer^26^, and colorectal cancer^59^, again supporting the vital role of ENO1 as an oncogene that promotes tumor progression. Emerging data indicate that ENO1 silencing inhibits tumor cell proliferation and invasion and reverses EMT progression^60–63^. In our study, we investigated HCC growth and metastasis in the presence and absence of ENO1. Consistent with previous studies, our gain- and loss-of-function studies showed that ENO1 upregulation significantly promotes cell proliferation, migration, invasion, and EMT in HCC cells. In addition, a recent study showed that a high serum ENO1 level can predict the microvascular invasion of HCC^64^. We infer that ENO1 may be released into serum or tissues in an exosome-dependent manner, thereby performing its biological function and promoting tumor progression. Our study confirmed that ENO1 regulation in HCC cells can affect ENO1 expression in exosomes derived from these cells but does not affect the release or uptake of exosomes. Exosome education experiments showed that the uptake of exosome-derived ENO1 by HCC cells with low ENO1 expression to achieve horizontal transfer of ENO1. Exosome-derived ENO1 plays a role similar to upregulating intracellular ENO1, promoting cellular malignant transformation and lung metastasis of HCC cells with low ENO1 expression. Our study provides strong evidence that exosome-derived ENO1 is a key determinant of HCC growth and metastasis. Understanding the biological function of exosome-derived ENO1 and actualizing liquid biopsy techniques to assess exosomal ENO1 may have valuable implications for the clinical evaluation of HCC progression, especially the prediction of metastatic risk.

Although previous studies mainly regarded ENO1 as a glycolytic enzyme regulating the energy metabolism in tumor cells to promote tumor progression, recent studies have shown that in addition to some metabolism-related proteins, ENO1 silencing also regulates the expression of some cell cycle-, adhesion- and migration-related proteins, including integrin family members^24^. A previous study reported that ENO1 controls alpha v/beta 3 integrin expression and regulates pancreatic cancer adhesion, invasion, and metastasis^26^. However, it is not yet clear which type of integrins can be regulated by ENO1 in HCC cells. In our study, we found that ENO1 regulated integrin α1, α2, α6, αV, and β4 expression in HCC cells. Among them, integrin α6 and β4 were most significantly altered by changes in ENO1 expression. Surprisingly, exosome-derived ENO1 can also upregulate integrin α6β4 expression in recipient cells, which exhibit increased proliferative activity, migratory and invasive capacities, and EMT activation. Integrin α6β4 is a crucial protein complex involved in the spreading and metastasis of tumor cells^65,66^. Integrin α6β4 separates from hemidesmosome-like structures in tumors, and this separation not only causes tumor cells to lose polarity and detach from the basement membrane but also causes upregulation of free integrin α6β4, which functions in bidirectional signal transduction and can, in turn, activate the relevant signaling pathways to promote tumor growth and metastasis^67,68^. Under conditions of hemidesmosome disassembly, integrin α6β4 can activate multiple signal transduction cascades either by directly binding its ligand laminin or by cooperating with multiple different growth factor receptors, including EGFR, ErbB2, c-Met, Ron, and LPAR^68^. The primary downstream signaling cascade involves the activation of FAK, Src, PI3K, AKT, MAPK, and the Rho small GTPase pathways^69–72^. After education with exosome-derived ENO1, we observed the clustering of integrin α6β4, and the activation of FAK and Src in HCC cells. Abnormally elevated activity of FAK and Src causes pleiotropic cellular responses inducing cell proliferation, transformation, and metastasis^73,74^, phenomena that completely align with our study. In addition, we also observed the phosphorylation of p38MAPK at the same time. p38MAPKs are activated by environmental and genotoxic stresses and play key roles in inflammation, as well as in tissue homeostasis, as they control cell proliferation, differentiation and survival and the migration of specific cell types^75^. Evidence indicates that p38MAPK activation is required for the invasive capacity of HCC, and is associated with poor survival in HCC patients^76,77^. These results suggest that the exosomes with high ENO1 expression in the extracellular environment may act as autocrine/paracrine stimuli in a heterogeneous tumor cell population to promote HCC growth and metastasis.

**Fig. 7:**
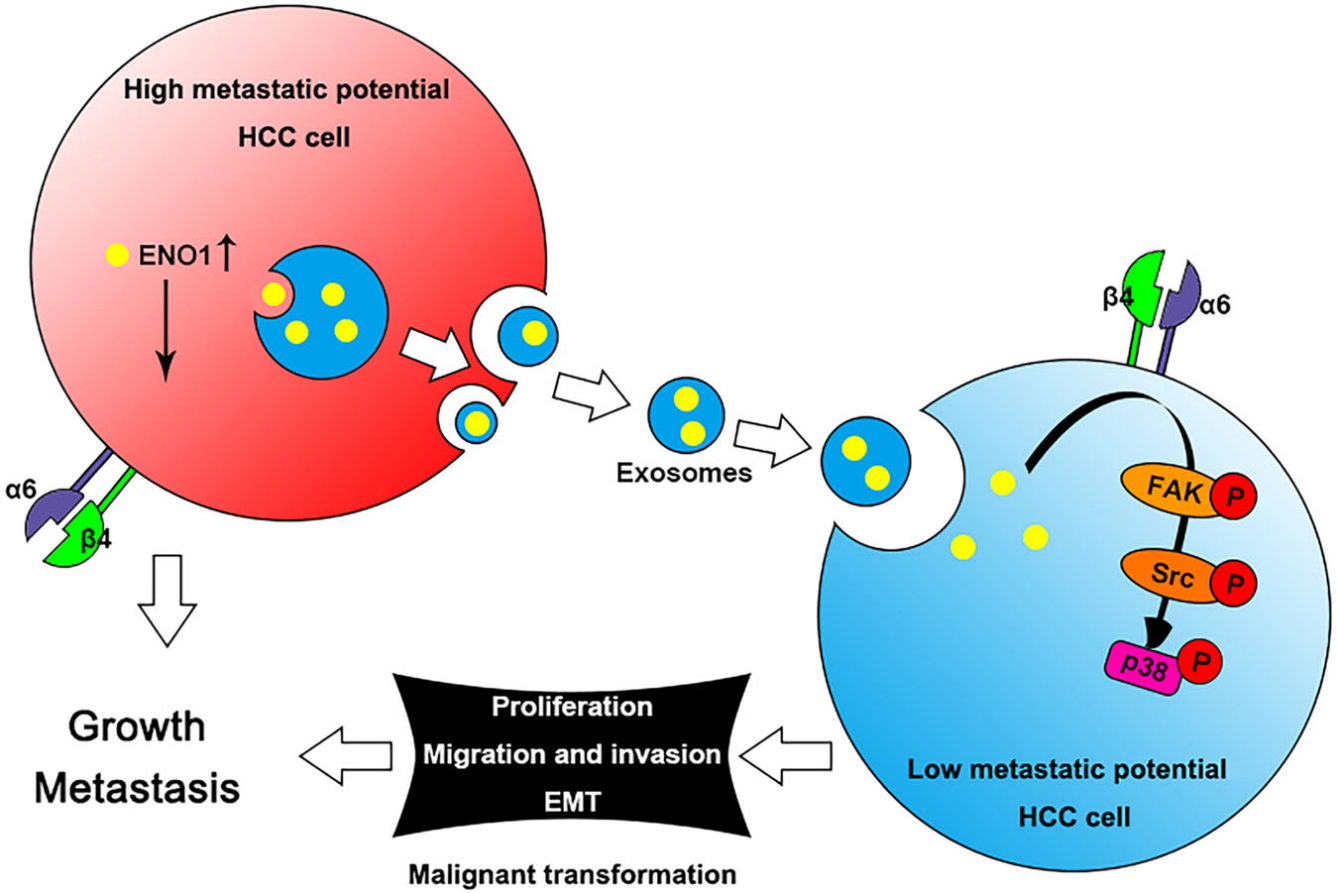
Schematic diagram of the role of exosome-derived ENO1 in HCC growth and metastasis. Exosome-derived ENO1 facilitates the expression of integrin α6β4 and the activation of the FAK/Src pathway, supporting downstream signaling via p38MAPK in HCC cells.

In conclusion, we provide the evidence that the tumor-promoting properties of ENO1 can be transferred between HCC cells with high/low ENO1 expression via exosome-mediated crosstalk, promoting the growth and metastasis of HCC by upregulating integrin α6β4 expression and activating the FAK/Src-p38MAPK pathway (Fig. 7). However, our current study does have some limitations. Although ENO1 is not limited to a single glycolytic enzyme function and the nonmetabolic role of exosome-derived ENO1 is also the key to promoting the growth and metastasis of HCC, it is difficult to exclude the effect of exosome-derived ENO1 on glycolysis in recipient cells. Therefore, it is necessary to further reveal the specific mechanism of exosomes-derived ENO1 regulation of integrin α6β4 expression to gain a deeper understanding of the role of exosomes-derived ENO1 in the growth and metastasis of HCC.

## Supplementary information

Supplementary Figure 1

Supplementary Figure 2

Supplementary Figure 3

Supplementary Figure 4

Supplementary Figure 5

Supplementary Figure 6
