# Supplementary figures and images for: Exosome-derived ENO1 regulates integrin α6β4 expression and promotes hepatocellular carcinoma growth and metastasis
Source: Cell Death Dis. 2020 Nov 12;11(11):972. doi: 10.1038/s41419-020-03179-1 (PMC7661725; doi:10.1038/s41419-020-03179-1)

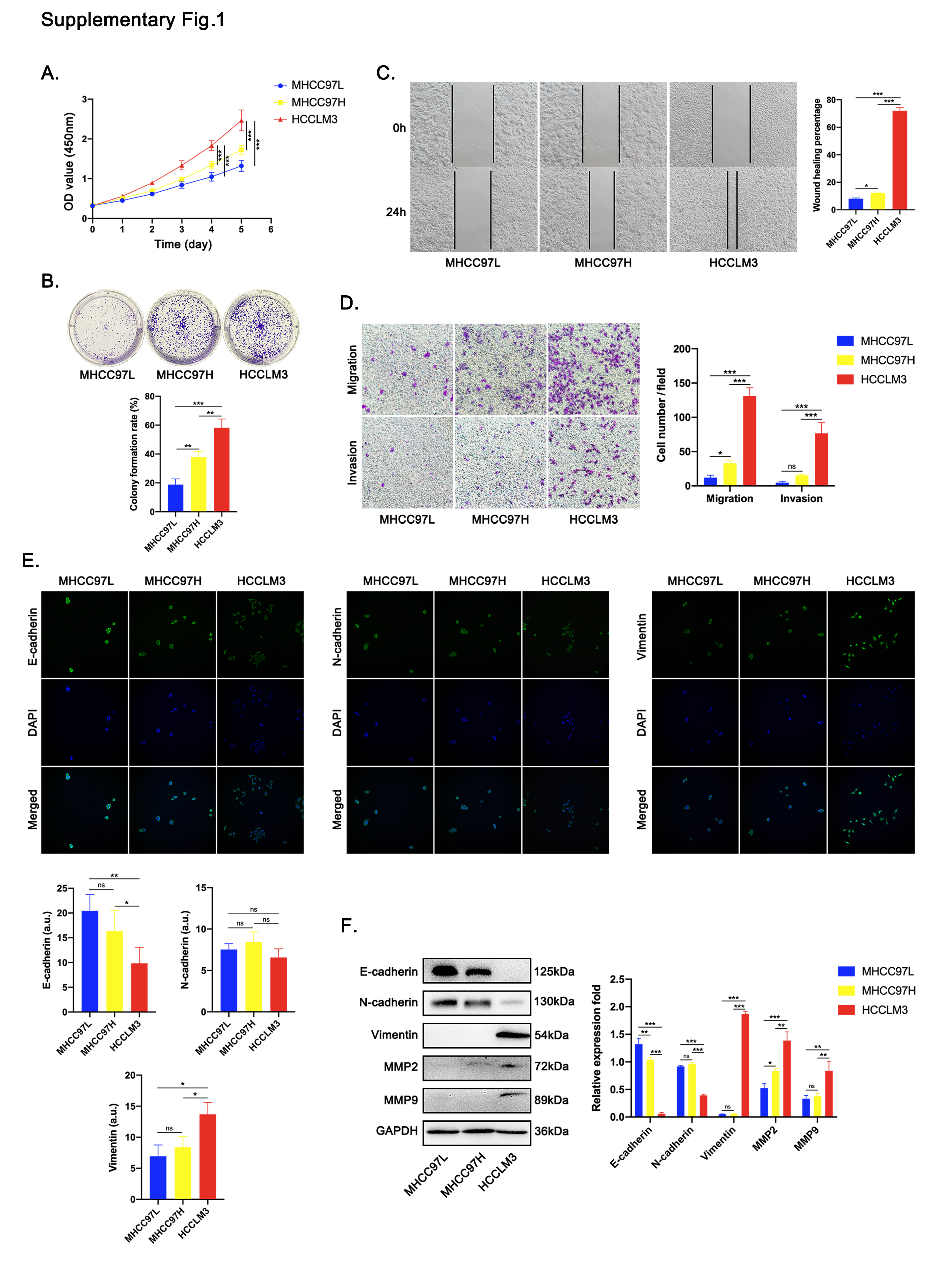

Supplement: Supplementary file 1 — Supplementary Figure 1 [file 41419_2020_3179_MOESM1_ESM.jpg]

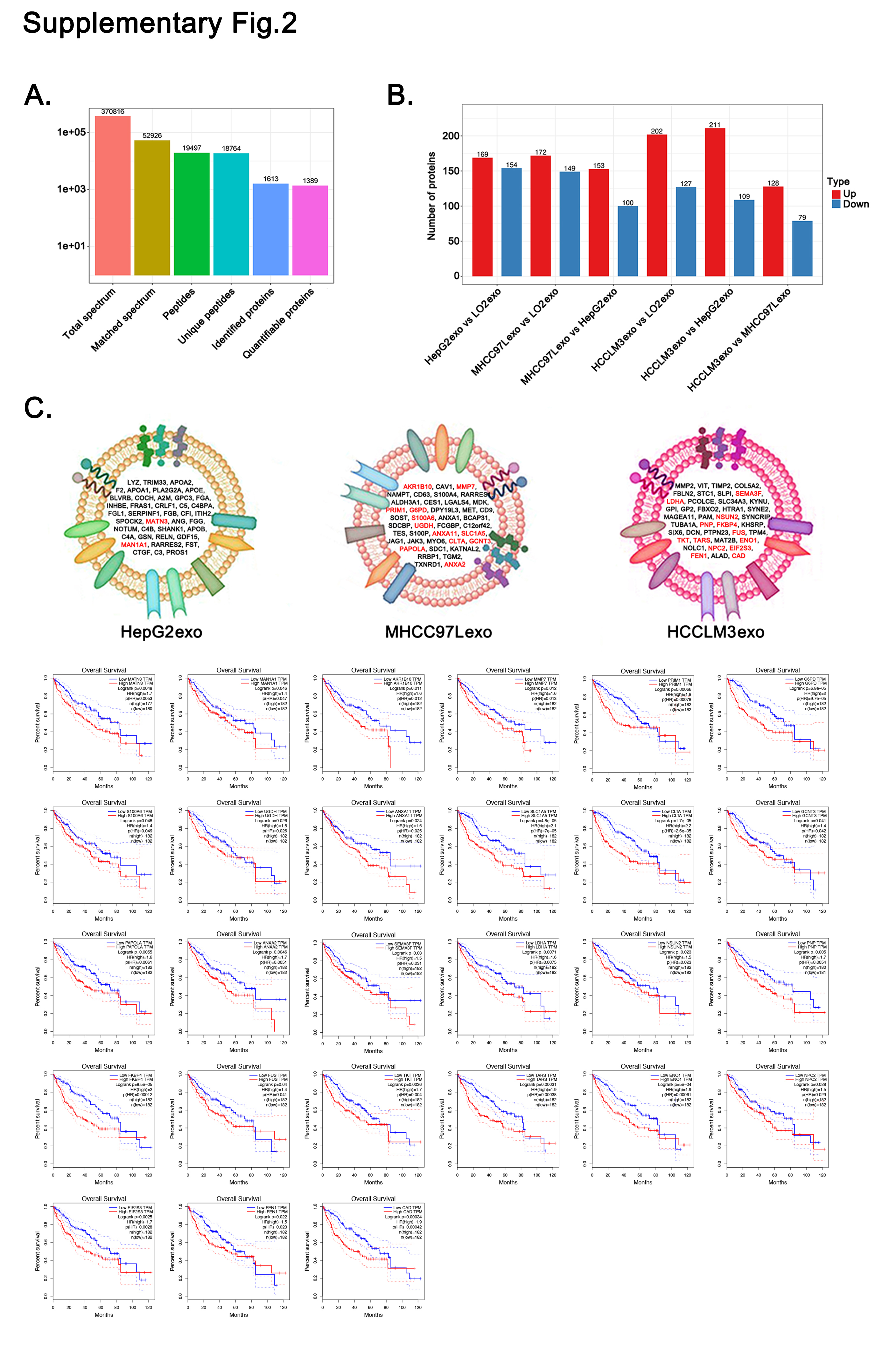

Supplement: Supplementary file 2 — Supplementary Figure 2 [file 41419_2020_3179_MOESM2_ESM.jpg]

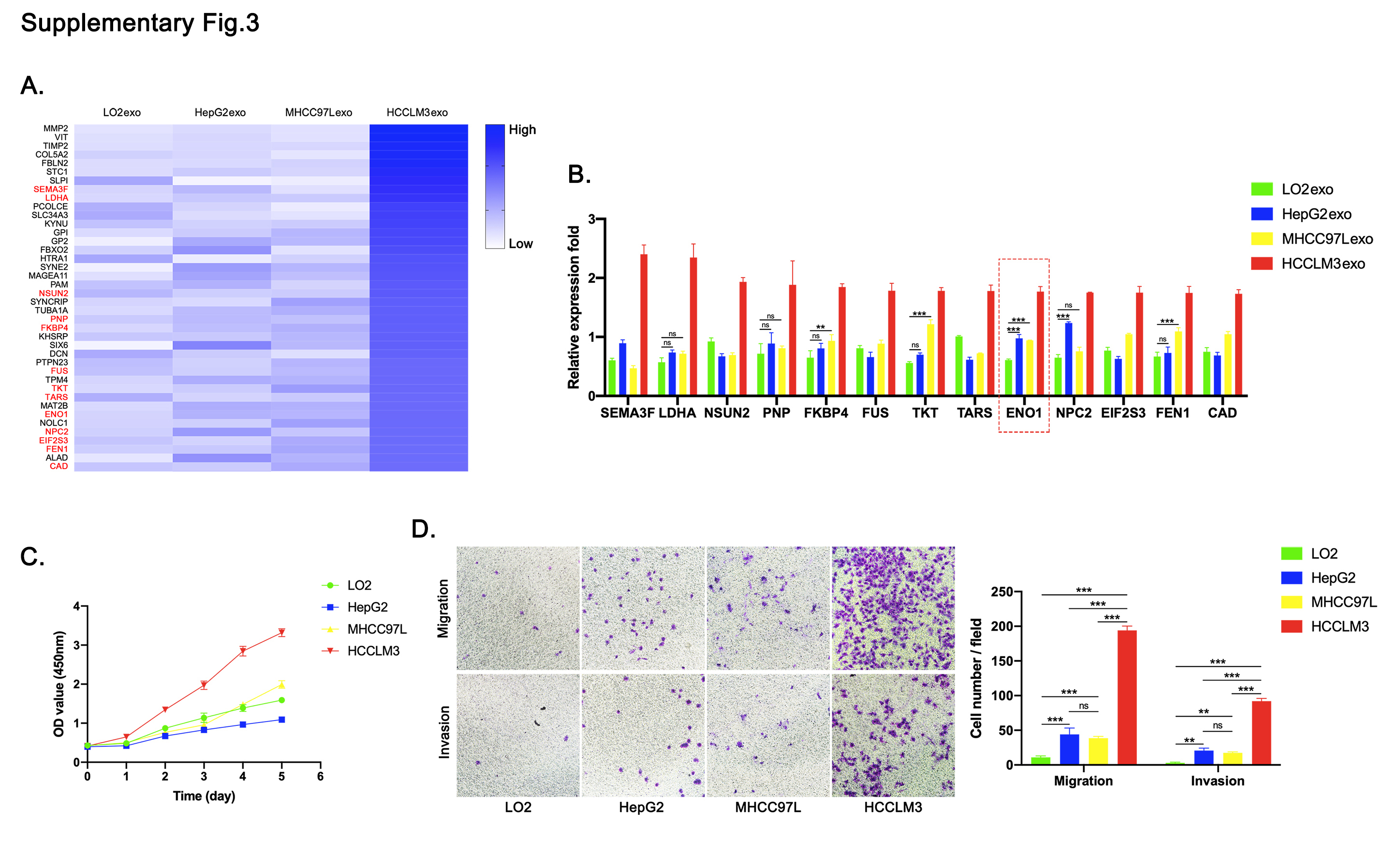

Supplement: Supplementary file 3 — Supplementary Figure 3 [file 41419_2020_3179_MOESM3_ESM.jpg]

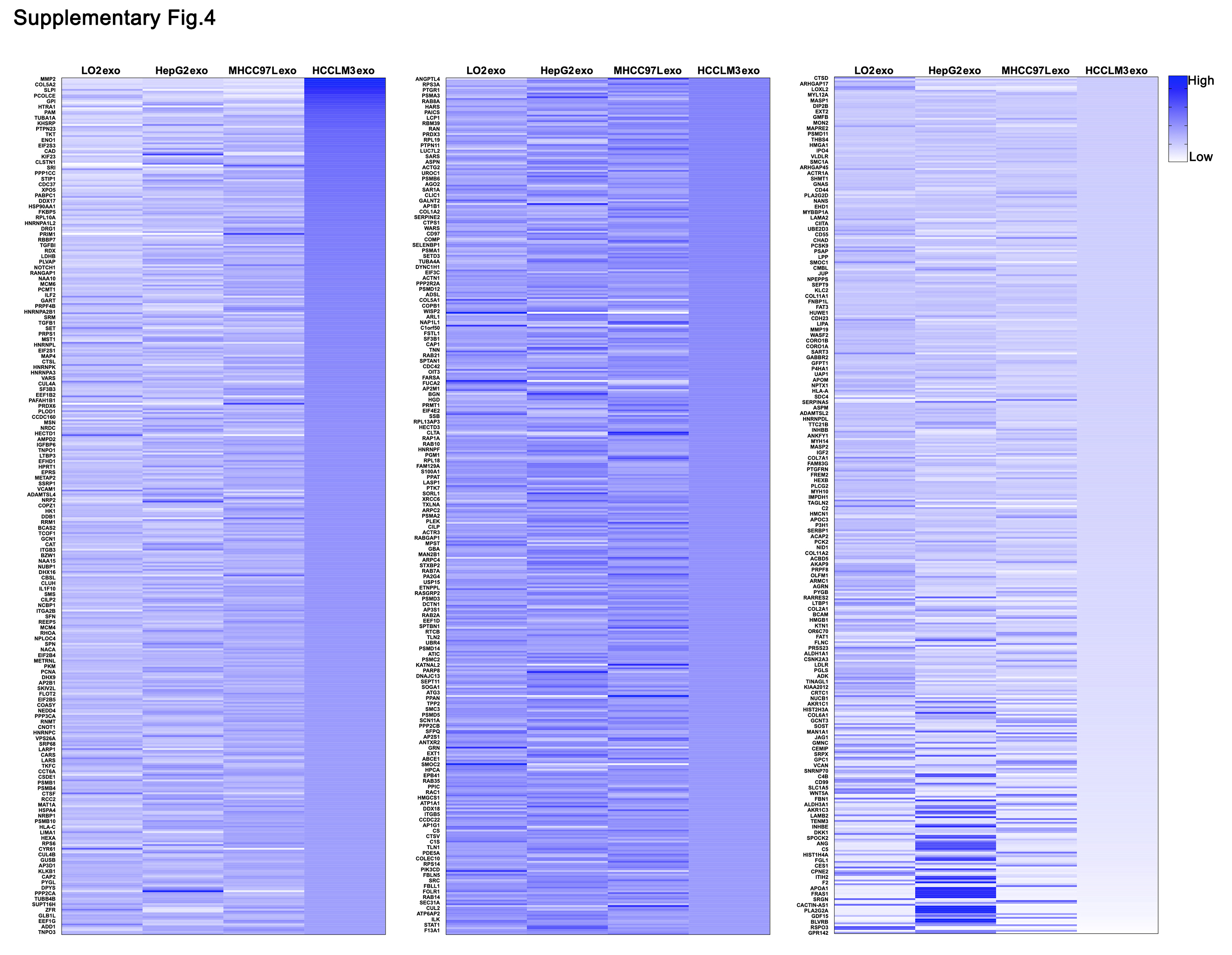

Supplement: Supplementary file 4 — Supplementary Figure 4 [file 41419_2020_3179_MOESM4_ESM.jpg]

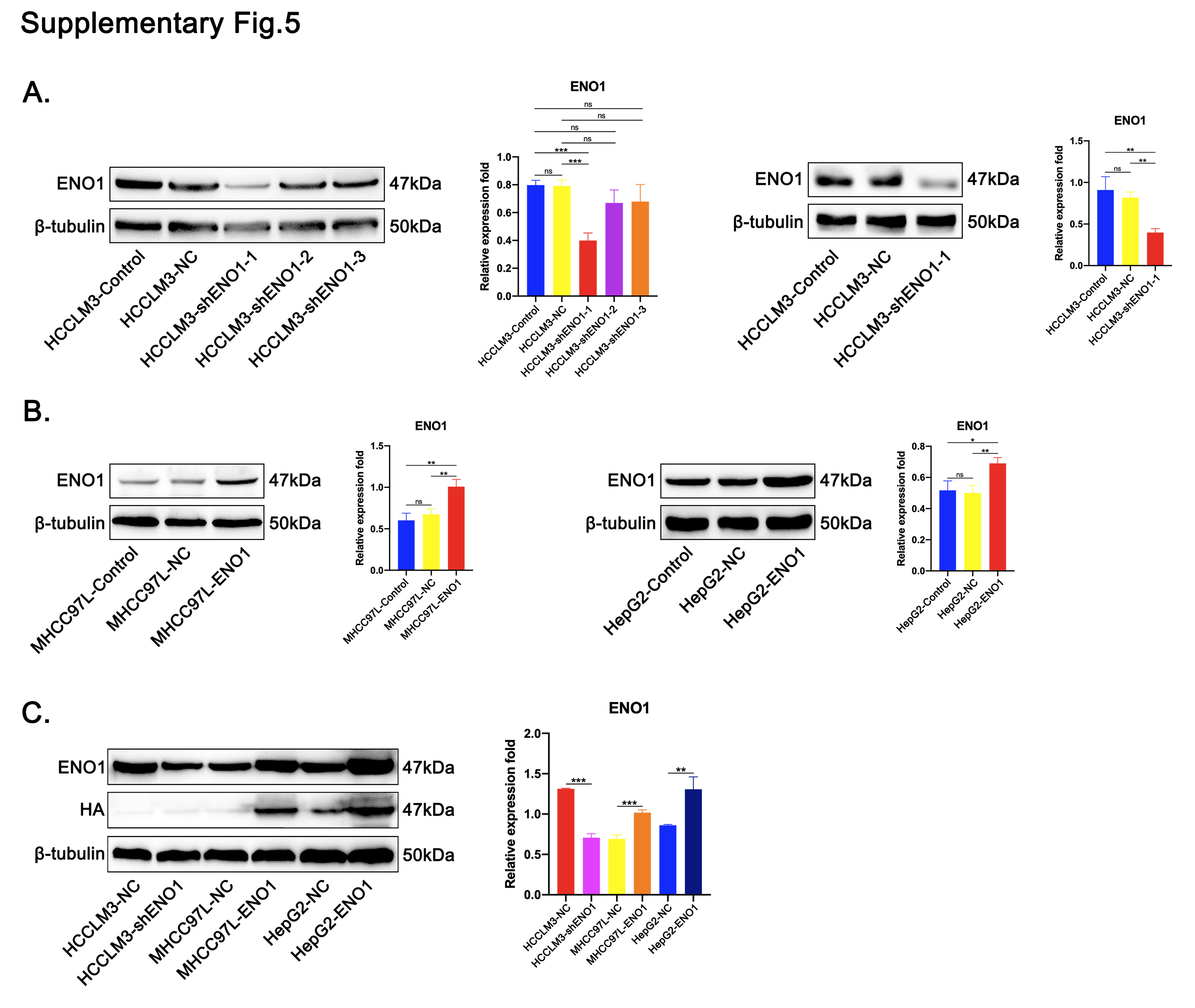

Supplement: Supplementary file 5 — Supplementary Figure 5 [file 41419_2020_3179_MOESM5_ESM.jpg]

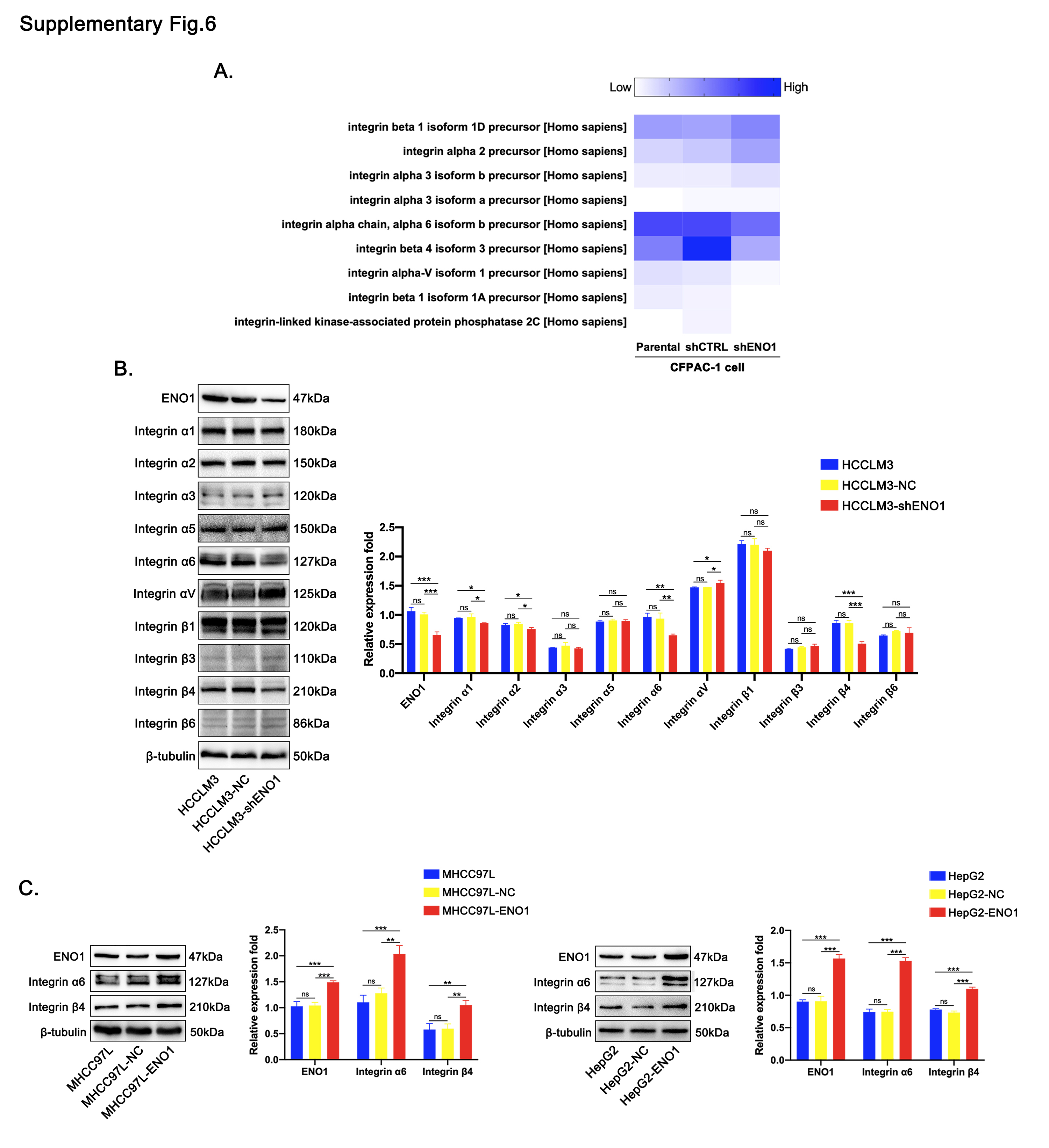

Supplement: Supplementary file 6 — Supplementary Figure 6 [file 41419_2020_3179_MOESM6_ESM.jpg]
